# Anti-psychotic drugs act synergistically in combination with antifungal drugs to inhibit drug-resistant *Cryptococcus neoformans* and *Candida albicans*

**DOI:** 10.1128/msphere.00432-26

**Published:** 2026-09-10

**Authors:** Magali Ayala, Alifia Jakamartana, Rodrigo Mamede Dos Santos Costa, Eric Cheuk-Kiu Cheung, Joseph M. Bednarek, Christian T. Moreau, Jessica C. S. Brown

**Affiliations:** 1 School of Biological Sciences, University of Utah 172838, Salt Lake City, Utah, USA; University of Guelph, Guelph, Ontario, Canada

**Keywords:** fungal infections, drug resistance, antifungal resistance, lipid biosynthesis

## Abstract

Systemic fungal infections cause an estimated 3.8 million deaths annually, approximately 10% of which are caused by drug-resistant infections. With only five classes of antifungal drugs, treatment options are limited. Here, we explore synergistic drug combinations—when the efficacy of two drugs combined is greater than expected based on the sum of each individual drug’s efficacy—to improve treatment of drug-resistant *Cryptococcus neoformans* and *Candida albicans*. Chlorpromazine acts synergistically with both amphotericin B and fluconazole against multiple fungal species, including azole-resistant *C. neoformans* and *C. albicans*. We then performed a genome-wide knockout mutant screen and found that ESCRT pathway mutants are resistant to chlorpromazine, while knockout mutants of genes involved in fatty acid biosynthesis are sensitive. Based on these data, we investigated sterol and fatty acid composition in chlorpromazine-treated cells and found only minor increases in sterol precursors, but a substantial increase in lipid droplet size and decreased lipid droplet numbers. This lipid droplet formation potentially sequesters lipid bioavailability and response to membrane stress. Together, these data suggest that chlorpromazine and its analogs are potentially promising treatments for systemic fungal infections that act via lipid homeostasis and stress response.

**IMPORTANCE:**

Fungal infections are a large and expensive health burden with high mortality rates. People with compromised immune systems from cancer, solid organ transplant, HIV infection, and other conditions are particularly affected. Systemic fungal infections are difficult to treat because there are few available drugs and treatment periods last months or years. Long treatment times increase the risk of treatment failure and can contribute to the rise of resistance. We identified an additional class of drugs, chlorpromazine and other phenothiazine drugs, that amplify the activity of existing antifungal drugs amphotericin B (AmB) and fluconazole (FLZ). AmB and FLZ act by targeting ergosterol, the fungal equivalent of cholesterol, which is required for a functional plasma membrane. Chlorpromazine increases the formation of lipid drops, which sequester lipids such as ergosterol. When chlorpromazine is combined with AmB, the fungal cell cannot respond to the plasma membrane damage caused by AmB, inhibiting the fungal cells. This work identifies new target processes and drugs that could treat deadly fungal infections.

## INTRODUCTION

Human interactions with environmental fungi are common but mostly benign: every time we breathe, we inhale fungal spores (1). However, a small subset of species causes severe, systemic disease. These infections have a high mortality rate, causing an estimated 3.8 million deaths annually (2). There are only five available drug classes to treat systemic fungal infections (3): (i) polyenes (e.g., amphotericin B [AmB]) (4, 5) and (ii) azoles (e.g., fluconazole [FLZ]) (6), which target the plasma membrane sterol ergosterol and its synthesis; (iii) nucleoside analogs that target DNA and RNA synthesis, which can only be used in combination with other therapies (7); (iv) echinocandins, which target β-1,3-D-glucan synthase in the cell wall (8); and (v) ibrexafungerp (9), the first member of the triterpenoid class, which acts on the same target as echinocandins (10). Due to the increasing incidence and severity of fungal diseases, in 2020, the World Health Organization published its first fungal disease priority list (11). *Cryptococcus neoformans*, the primary cause of fungal meningitis and the second most common cause of AIDS-related mortality (12), was one of four critical priority status fungal pathogens. Approximately 152,000 cryptococcal meningitis cases were reported in 2020, with an estimated 112,000 deaths (2). *C. neoformans* infections disproportionately affect the immunocompromised, particularly those with low CD4^+^ T-cell counts (13, 14). However, non-HIV cryptococcosis cases are growing: new estimates reported 22,280 cases in individuals with no apparent underlying disease (2).

The mortality rate from cryptococcal infection varies from 24% to 75% with treatment availability (2, 15). In low-income settings, treatment is particularly challenging due to insufficient resources and difficulty managing the disease. The standard therapy for cryptococcal meningitis consists of induction treatment with a single dose of liposomal amphotericin B and 2 weeks of 5-fluorocytosine (5-FC) (nucleoside analog) or fluconazole. This is followed by consolidation (8 weeks) and maintenance (12 months) treatment with fluconazole. When AmB is unavailable, the alternative treatment is fluconazole monotherapy or a combination of fluconazole plus 5-FC; both alternatives increase the mortality rate (15). The long treatment duration requires a high degree of patient compliance and carries the potential for side effects such as nephrotoxicity, hepatotoxicity, and bone marrow changes (7, 16).

Combination therapy using a repurposed drug along with an existing antifungal drug is a promising alternative approach to treating systemic fungal infections (17). New drug development is costly and complex (18), whereas repurposing allows off-label usage of drugs approved for other indications, allowing bypass of costly clinical trials. Here, we investigate synergistic combinations, where the efficacy of two drugs combined is greater than the sum of their individual effects (19). Synergistic drug combinations can increase therapeutic success when monotherapy fails to achieve the desired results. For example, the use of single antifungals such as fluconazole for the treatment of fungal infections is associated with the emergence of antifungal resistance (20). Synergistic drug interactions can prevent the development of resistance or combat it by targeting two key proteins in the same pathway or in two separate pathways (21–24).

Drugs not specifically developed as antifungal drugs have been shown to enhance antifungal potency when used in combination with an antifungal drug. A classic example is calcineurin inhibitors, which act synergistically with azoles and echinocandins (25–27). Multiple groups previously identified commonly prescribed drugs from a wide range of bioactive categories (anti-infective, antidepressants, antineoplastic, among others) that are synergistic with fluconazole against *C. neoformans* by unknown mechanisms (24, 28–31). We additionally identified that the antipsychotic drug chlorpromazine acts synergistically when combined with fluconazole (28) or AmB against *C. neoformans* and *Candida albicans*. The goal of this study is to identify the molecular mechanism underlying these synergistic interactions.

Chlorpromazine is a commonly prescribed first-generation phenothiazine antipsychotic (32) with amphiphilic properties (Fig. S1) (33). It crosses the blood-brain barrier and is primarily used to treat schizophrenia and bipolar disorder (32). Due to chlorpromazine’s dual hydrophobic and hydrophilic regions, it is thought to interact with phospholipids in membrane bilayers (34). Furthermore, because it is widely available and relatively inexpensive, it is an attractive candidate for treating cryptococcal meningitis and other diseases caused by disseminating fungal pathogens. Previous studies showed that chlorpromazine and other phenothiazines inhibit the growth of pathogenic fungi (35). Another phenothiazine, trifluoperazine, binds *C. neoformans* calmodulin *in vitro* (36). Calmodulin is a highly connected protein with many interacting partners that acts in multiple calcium-dependent and -independent pathways important for *C. neoformans* biology and pathogenesis (37, 38).

Changes in membrane composition affect membrane fluidity (39), transport processes (40), lipid-protein interactions (41, 42), and cell signaling (43). All these processes influence lipid biosynthesis (44). Lipid biosynthesis and function are also common antifungal drug targets. Ergosterol, the fungal analog of mammalian cholesterol, is essential in fungi (45). AmB increases membrane permeability and reactive oxygen species by binding and extracting ergosterol from the plasma membrane in *C. neoformans* (46, 47), while triazole antifungals target 14α-demethylase (Erg11) in the ergosterol biosynthesis pathway (48).

In this study, we elucidate the molecular mechanisms of the AmB and chlorpromazine combination and their synergistic interaction against the fungal pathogen *C. neoformans*. This combination has been effective against >90% of clinical isolates and demonstrated synergy against *Candida albicans*, including clinical isolates. We identify chlorpromazine’s interference with lipid homeostasis. Together, these data demonstrate that disrupting ergosterol homeostasis (with AmB or fluconazole) and inducing large lipid droplet formation (chlorpromazine) act synergistically to inhibit fungal cells.

## RESULTS

### Chlorpromazine acts synergistically with multiple antifungals against *Candida albicans* and *C. neoformans* clinical isolates

**Fig 1:**
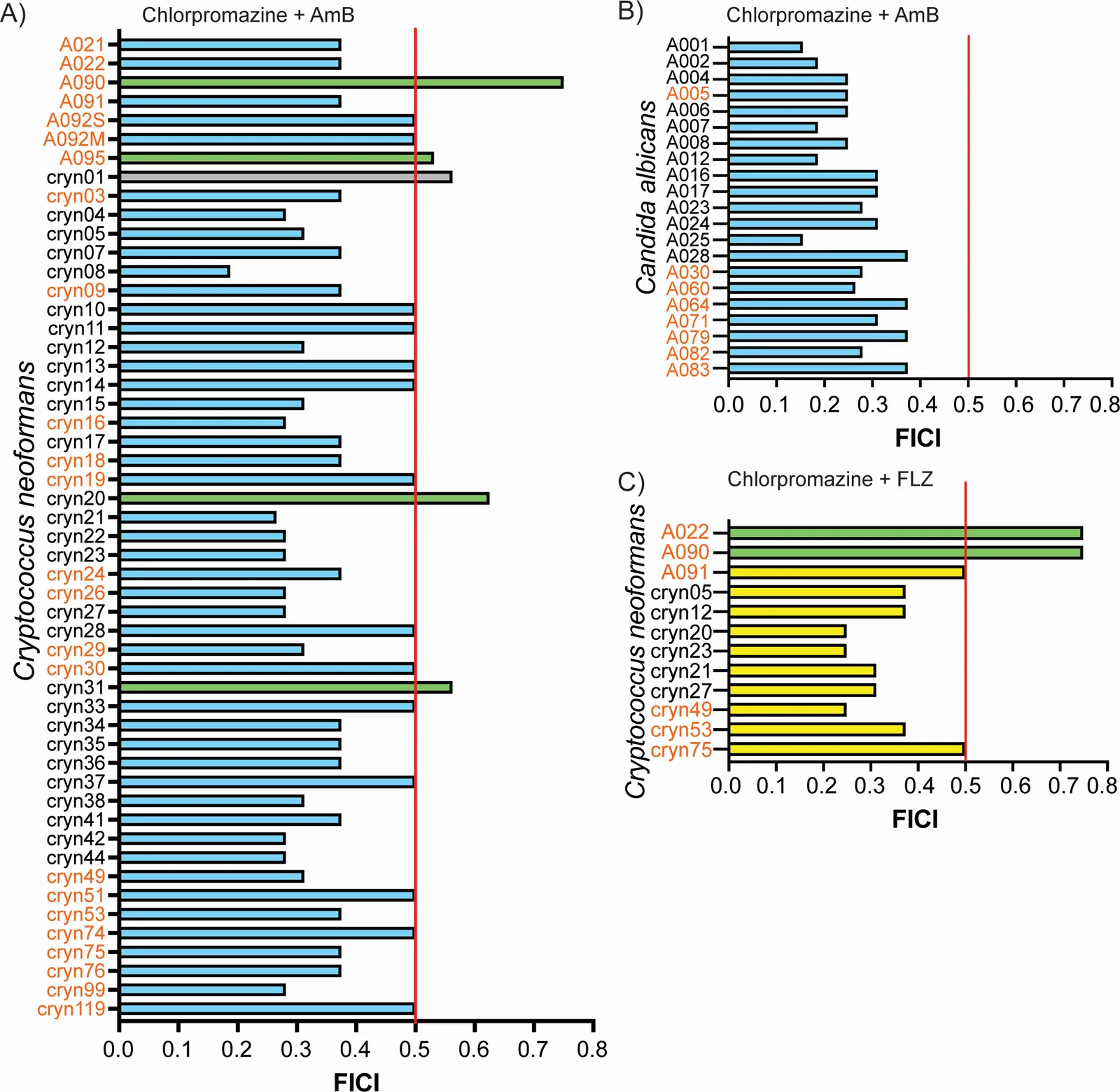
Synergistic effect of chlorpromazine in combination with amphotericin B or fluconazole in multiple clinical isolates and different fungal strains. FICI scores of (**A**) *C. neoformans* clinical isolates exposed to chlorpromazine and AmB, (**B**) *C. albicans* clinical isolates exposed to chlorpromazine and AmB, and (**C**) *C. neoformans* clinical isolates exposed to chlorpromazine and fluconazole. Blue (**A and B**) and yellow (**C**) bars represent an FICI score of ≤0.5, indicating a synergistic response to the two drugs. Gray bars represent an indifferent interaction (less than the sum of the inhibition caused by each drug), and green bars represent an additive interaction (greater than or equal to the sum of the inhibition caused by each drug but less than the fourfold increase in the inhibition of each drug required for a synergistic interaction). The red vertical line marks the division between synergistic and non-synergistic at the 0.5 mark. Names of strains resistant or susceptible-dose dependent are labeled in orange.

We first investigated the breadth of the synergistic interaction between chlorpromazine and the antifungals AmB or fluconazole. We chose multiple clinical isolates of *C. neoformans* and *C. albicans* with varying levels of fluconazole resistance and susceptibility (Table 1) and tested for synergy using checkerboard assays. Synergy was calculated using the fractional inhibitory concentration index (FICI), where an FICI ≤0.5 indicates synergy (49), corresponding to at least a fourfold reduction in the minimum inhibitory concentration (MIC) of each drug in combination. The AmB and chlorpromazine combination acted synergistically against 47 of the 52 *C. neoformans* clinical isolates tested, including 22 of the 24 fluconazole-resistant isolates (Fig. 1A; Table 2). Chlorpromazine in combination with AmB also acts synergistically against *Candida albicans* clinical isolates: all tested isolates, including eight fluconazole-resistant isolates, showing an FICI of <0.4 (Fig. 1B; Table 3). None of the isolates exhibited an antagonistic response (when one drug inhibits another’s activity). When the response was not synergistic, it was additive, with the combination’s activity approximately equal to the sum of each individual drug’s activity. We did not observe indifference. Together, these data suggest that there are no detrimental effects of combining chlorpromazine and AmB as far as the response of *C. neoformans* isolates is concerned.

**TABLE 1:** List of *C. neoformans* clinical isolates resistant and susceptible to fluconazole

Name	FLZ susceptibility	MIC (µg/mL)
A022	Susceptible-dose dependent	16
A090	Susceptible-dose dependent	16
A091	Susceptible-dose dependent	16
A092M	Susceptible-dose dependent	32
A092S	Susceptible-dose dependent	32
A095	Susceptible-dose dependent	16
cryn01	Susceptible	1
cryn02	Susceptible-dose dependent	32
cryn03	Susceptible-dose dependent	16
cryn04	Susceptible	8
cryn05	Susceptible	4
cryn07	Susceptible	8
cryn08	Susceptible	2
cryn09	Resistant	64
cryn10	Susceptible	2
cryn11	Susceptible	4
cryn12	Susceptible	4
cryn13	Susceptible	4
cryn14	Susceptible	2
cryn15	Susceptible	4
cryn16	Susceptible-dose dependent	16
cryn17	Susceptible	4
cryn18	Susceptible-dose dependent	16
cryn19	Susceptible-dose dependent	16
cryn20	Susceptible	4
cryn21	Susceptible	8
cryn22	Susceptible	4
cryn23	Susceptible	8
cryn24	Susceptible-dose dependent	16
cryn26	Susceptible-dose dependent	16
cryn27	Susceptible	4
cryn28	Susceptible	2
cryn29	Susceptible-dose dependent	16
cryn30	Susceptible-dose dependent	16
cryn31	Susceptible	1
cryn33	Susceptible	2
cryn34	Susceptible	4
cryn35	Susceptible	4
cryn36	Susceptible	8
cryn37	Susceptible	2
cryn38	Susceptible	4
cryn41	Susceptible	4
cryn42	Susceptible	4
cryn44	Susceptible	4
cryn49	Susceptible-dose dependent	16
cryn51	Susceptible-dose dependent	16
cryn53	Susceptible-dose dependent	16
cryn74	Susceptible-dose dependent	16
cryn75	Susceptible-dose dependent	16
cryn76	Susceptible-dose dependent	16
cryn99	Susceptible-dose dependent	16
cryn119	Susceptible-dose dependent	32

**TABLE 2:** FICI scores and MICs of *C. neoformans* treated with chlorpromazine and AmB combination

Species	Strain name	CPZ MIC (µg/mL)	AmB MIC (µg/mL)	FICI score
*Cryptococcus neoformans*	A021	88	5	0.375
*Cryptococcus neoformans*	A022	88	5	0.375
*Cryptococcus neoformans*	A090	175	2.5	0.75
*Cryptococcus neoformans*	A091	175	2.5	0.375
*Cryptococcus neoformans*	A092S	175	2.5	0.5
*Cryptococcus neoformans*	A092M	88	2.5	0.5
*Cryptococcus neoformans*	A095	88	1.25	0.531
*Cryptococcus neoformans*	cryn01	350	5	0.563
*Cryptococcus neoformans*	cryn03	350	5	0.375
*Cryptococcus neoformans*	cryn04	175	2.5	0.281
*Cryptococcus neoformans*	cryn05	175	5	0.313
*Cryptococcus neoformans*	cryn07	175	2.5	0.375
*Cryptococcus neoformans*	cryn08	175	2.5	0.188
*Cryptococcus neoformans*	cryn09	44	5	0.375
*Cryptococcus neoformans*	cryn10	88	1.25	0.5
*Cryptococcus neoformans*	cryn11	175	2.5	0.5
*Cryptococcus neoformans*	cryn12	88	2.5	0.313
*Cryptococcus neoformans*	cryn13	44	2.5	0.5
*Cryptococcus neoformans*	cryn14	88	1.25	0.5
*Cryptococcus neoformans*	cryn15	175	2.5	0.313
*Cryptococcus neoformans*	cryn16	175	2.5	0.281
*Cryptococcus neoformans*	cryn17	175	2.5	0.375
*Cryptococcus neoformans*	cryn18	175	2.5	0.375
*Cryptococcus neoformans*	cryn19	175	0.625	0.5
*Cryptococcus neoformans*	cryn20	175	0.625	0.625
*Cryptococcus neoformans*	cryn21	88	2.5	0.266
*Cryptococcus neoformans*	cryn22	88	2.5	0.281
*Cryptococcus neoformans*	cryn23	88	2.5	0.281
*Cryptococcus neoformans*	cryn24	44	2.5	0.375
*Cryptococcus neoformans*	cryn26	88	2.5	0.281
*Cryptococcus neoformans*	cryn27	88	2.5	0.281
*Cryptococcus neoformans*	cryn28	175	5	0.5
*Cryptococcus neoformans*	cryn29	44	1.25	0.313
*Cryptococcus neoformans*	cryn30	175	2.5	0.5
*Cryptococcus neoformans*	cryn31	175	1.25	0.563
*Cryptococcus neoformans*	cryn33	175	5	0.5
*Cryptococcus neoformans*	cryn34	44	5	0.375
*Cryptococcus neoformans*	cryn35	88	2.5	0.375
*Cryptococcus neoformans*	cryn36	88	2.5	0.375
*Cryptococcus neoformans*	cryn37	175	2.5	0.5
*Cryptococcus neoformans*	cryn38	88	2.5	0.313
*Cryptococcus neoformans*	cryn41	88	5	0.375
*Cryptococcus neoformans*	cryn42	88	5	0.281
*Cryptococcus neoformans*	cryn44	88	5	0.281
*Cryptococcus neoformans*	cryn49	175	5	0.313
*Cryptococcus neoformans*	cryn51	350	5	0.5
*Cryptococcus neoformans*	cryn53	350	2.5	0.375
*Cryptococcus neoformans*	cryn74	175	2.5	0.5
*Cryptococcus neoformans*	cryn75	175	2.5	0.375
*Cryptococcus neoformans*	cryn76	88	2.5	0.375
*Cryptococcus neoformans*	cryn99	88	2.5	0.281
*Cryptococcus neoformans*	cryn119	175	2.5	0.5
*Cryptococcus neoformans*	KN99	88	2.5	0.5

**TABLE 3:** FICI scores and MICs of *Candida albicans* treated with chlorpromazine and AmB combination

Species	Strain name	CPZ MIC (µg/mL)	AmB MIC (µg/mL)	FICI score
*Candida albicans*	A001	175	0.8	0.16
*Candida albicans*	A002	175	0.8	0.19
*Candida albicans*	A004	175	1.6	0.25
*Candida albicans*	A005	175	0.8	0.25
*Candida albicans*	A006	350	0.6	0.25
*Candida albicans*	A007	350	0.6	0.19
*Candida albicans*	A008	350	0.6	0.25
*Candida albicans*	A012	350	0.6	0.19
*Candida albicans*	A016	350	2.5	0.31
*Candida albicans*	A017	350	1.3	0.31
*Candida albicans*	A023	350	1.3	0.28
*Candida albicans*	A024	350	2.5	0.31
*Candida albicans*	A025	350	1.3	0.16
*Candida albicans*	A028	350	1.3	0.38
*Candida albicans*	A030L	350	1.3	0.28
*Candida albicans*	A060	350	1.3	0.27
*Candida albicans*	A064	350	1.3	0.38
*Candida albicans*	A071	350	1.3	0.31
*Candida albicans*	A079	350	2.5	0.38
*Candida albicans*	A082	350	1.3	0.28
*Candida albicans*	A083	175	2.5	0.38

To assess whether chlorpromazine would exhibit similar synergistic activity when combined with fluconazole, we tested 12 *C. neoformans* isolates, and 10 showed a synergistic response (Fig. 1C; Table 4). One isolate that did not show a synergistic response to chlorpromazine + fluconazole, A022, exhibited a synergistic response to chlorpromazine + AmB. Isolate cryn20 showed the reverse: a non-synergistic response to the chlorpromazine + AmB combination but a synergistic response to chlorpromazine + fluconazole (Fig. 1C). Although the mechanism conferring fluconazole resistance in these clinical isolates is unknown, fluconazole resistance is commonly associated with mutations in Erg11 (50–53) or drug transporters (54, 55), as well as aneuploidies (56–58).

**TABLE 4:** FICI scores and MICs of *C. neoformans* treated with chlorpromazine and fluconazole combination

Species	Strain name	CPZ MIC (µg/mL)	FLZ MIC (µg/mL)	FICI score
*Cryptococcus neoformans*	A022	88	30	0.75
*Cryptococcus neoformans*	A090	88	30	0.75
*Cryptococcus neoformans*	A091	175	15	0.5
*Cryptococcus neoformans*	cryn05	175	7.5	0.375
*Cryptococcus neoformans*	cryn12	88	7.5	0.375
*Cryptococcus neoformans*	cryn23	175	15	0.25
*Cryptococcus neoformans*	cryn20	175	7.5	0.25
*Cryptococcus neoformans*	cryn21	175	15	0.3125
*Cryptococcus neoformans*	cryn27	175	15	0.3125
*Cryptococcus neoformans*	cryn49	175	15	0.25
*Cryptococcus neoformans*	cryn53	175	30	0.375
*Cryptococcus neoformans*	cryn75	175	15	0.25

AmB is an effective broad-spectrum antifungal for which, until recently, resistance was rare due to fitness tradeoffs (59, 60). Only two of the fluconazole-resistant *C. neoformans* clinical isolates tested did not show a synergistic response to AmB + chlorpromazine (A090 and A095). These results show that chlorpromazine strongly inhibits fungal cell growth when combined with either AmB or fluconazole against multiple *C. neoformans* clinical isolates *in vitro*, including fluconazole-resistant strains. AmB + chlorpromazine also acted synergistically against *C. albicans* isolates.

### Chlorpromazine decreases lanosterol and affects transcript levels of genes involved in the ergosterol synthesis pathway

**Fig 2:**
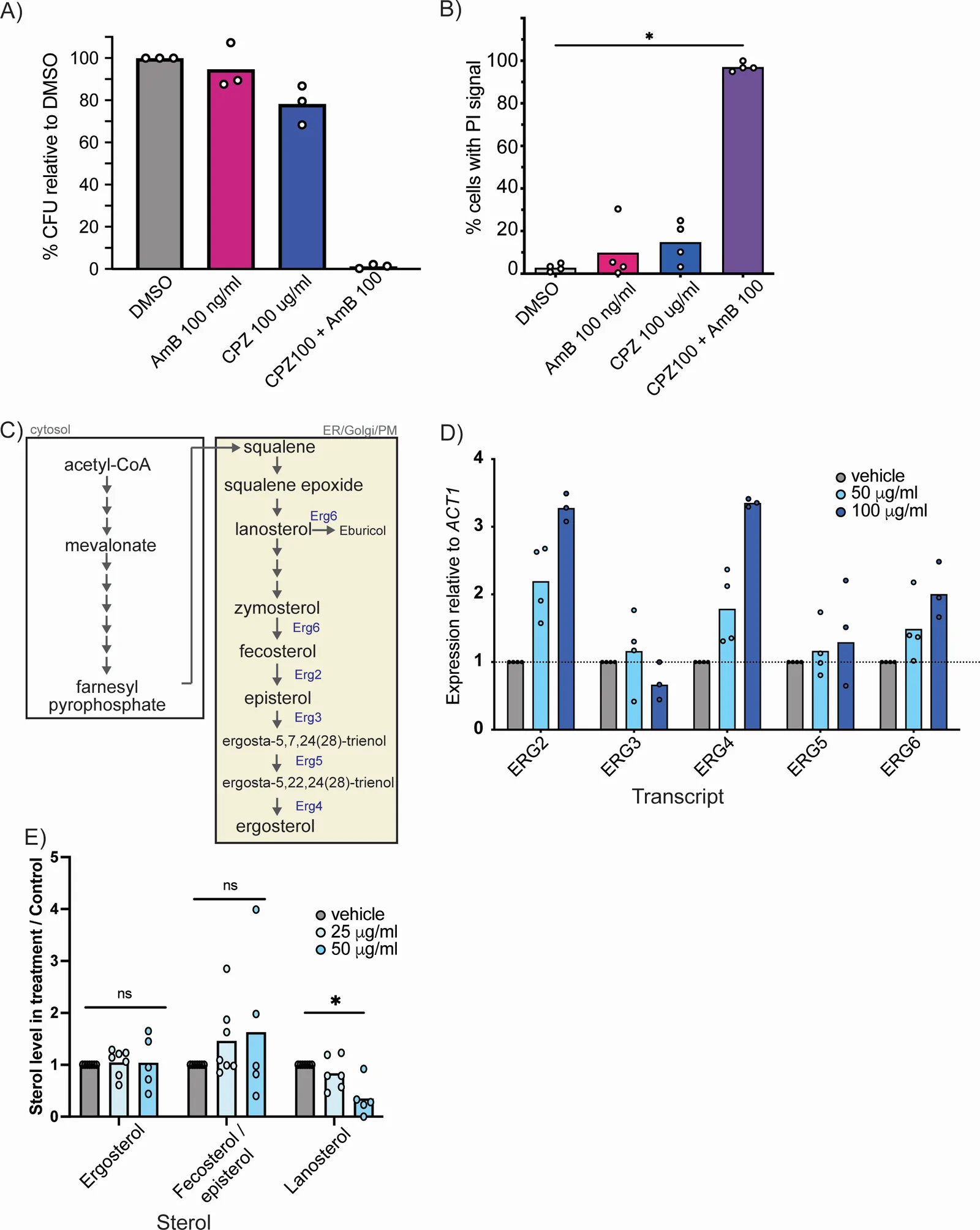
Chlorpromazine treatment increases *ERG2*, *ERG4*, and *ERG6* transcript levels in *C. neoformans*. (**A**) Percent viability of *C. neoformans* KN99 cells following a 3-h exposure to each drug. CFUs are normalized to the vehicle control. (**B**) Percent of cells positive for PI stain measured by flow cytometry after 3-h exposure to the small molecules listed. Each circle represents an individual biological replicate. (**C**) Ergosterol biosynthesis pathway, including *ERG* genes in blue. Steps occurring in the cytosol are in the left box and steps in the ER, Golgi, or plasma membrane in the right box. (**D**) Transcript levels normalized to *ACT1* and then normalized to cells treated with DMSO vehicle control. (**E**) Sterol quantification of cells treated with increasing chlorpromazine concentrations relative to cells treated with DMSO vehicle control. The same number of cells were harvested per sample, and samples were normalized to the vehicle control. We cannot distinguish the epimers fecosterol and episterol by mass spectrometry. Circles represent biological replicates. For all parts of this figure, *P < 0.05 and was calculated using the Mann-Whitney test.

Ergosterol is an abundant sterol important for fungal growth, and its biosynthetic pathway is the target of multiple antifungals (61). Sterols are synthesized in the endoplasmic reticulum (ER), then transported to the cell membranes to maintain membrane integrity (62). Due to chlorpromazine’s amphiphilic structure, it is hypothesized to insert into membrane bilayers by intercalating the hydrophobic head into the membranes (34), but its mechanism of action is unknown. We tested cell viability by exposing cells to concentrations of drugs that do not inhibit cell growth (100 ng/mL AmB or 100 μg/mL chlorpromazine) in the single-drug treatment, then measuring cell viability by colony-forming units (CFU) (Fig. 2A). The vehicle- and AmB-treated cells did not show any change in viability, and chlorpromazine-treated cells showed a modest 30% decrease in viability. In contrast, less than 2% of the cells in the AmB + CPZ treatment were alive, demonstrating that CPZ + AmB are fungicidal in combination. We also tested whether either AmB or chlorpromazine treatment damaged the plasma membrane, and these concentrations, since this would increase the bioavailability of drugs that need to enter the cell in order to act. Using propidium iodide (PI), which cannot cross the plasma membrane of living cells (63), neither AmB nor chlorpromazine significantly increased membrane permeability at these concentrations (Fig. 2B). However, when both drugs were combined, dye uptake increased fivefold, likely the result of dying cells.

We then considered that the increase in plasma membrane permeability could be an indirect result of chlorpromazine affecting membrane repair mechanisms (64). Since sterols are necessary to regulate plasma membrane permeability and fluidity (65), we focused on measuring the effects of chlorpromazine on ergosterol biosynthesis (Fig. 2C). We first examined the ergosterol biosynthesis pathway *ERG* genes via RT-qPCR. Transcript levels of *ERG2*, *ERG4*, and *ERG6*, late-stage genes in the pathway, significantly increased relative to the control (Fig. 2D). Genes early in the pathway are not upregulated (Fig. S2). Erg2 and Erg4 are localized to the ER in other fungi (66) but were not tested in *C. neoformans,* and Erg6 is localized to the ER and Golgi apparatus in *C. neoformans* (67). Ergosterol is synthesized and subsequently distributed to intracellular and plasma membranes from these locations (66).

Next, we investigated whether chlorpromazine directly affects ergosterol and its sterol pathway precursors by measuring sterol levels via mass spectrometry. After 24 h of chlorpromazine treatment, we found that levels of lanosterol, the first sterol after squalene (Fig. 2C), significantly decreased. However, levels of ergosterol and the sterols immediately upstream of ergosterol, fecosterol, and episterol do not change in response to chlorpromazine treatment (Fig. 2E). Since *ERG2*, *ERG4*, and *ERG6* transcripts are all upregulated in chlorpromazine-treated cells (Fig. 2D), we think that the later steps in ergosterol synthesis are increased, consuming lanosterol to produce sufficient ergosterol, but causing enough subcellular stress that chlorpromazine-treated cells cannot compensate for an additional stressor. Chlorpromazine might also be targeting the ergosterol biosynthesis pathway at multiple steps that have no net change in ergosterol. There might also be a feedback loop within the ergosterol pathway that upregulates downstream genes in response to changes in earlier sterols, as occurs in *Saccharomyces cerevisiae* (45). To distinguish between these, we screened two available (31, 68–70) *C. neoformans* single-gene knockout mutant libraries to search for sensitive or resistant mutants.

### Fatty acid biosynthesis genes and lipid droplet formation are important for the chlorpromazine response

**Fig 3:**
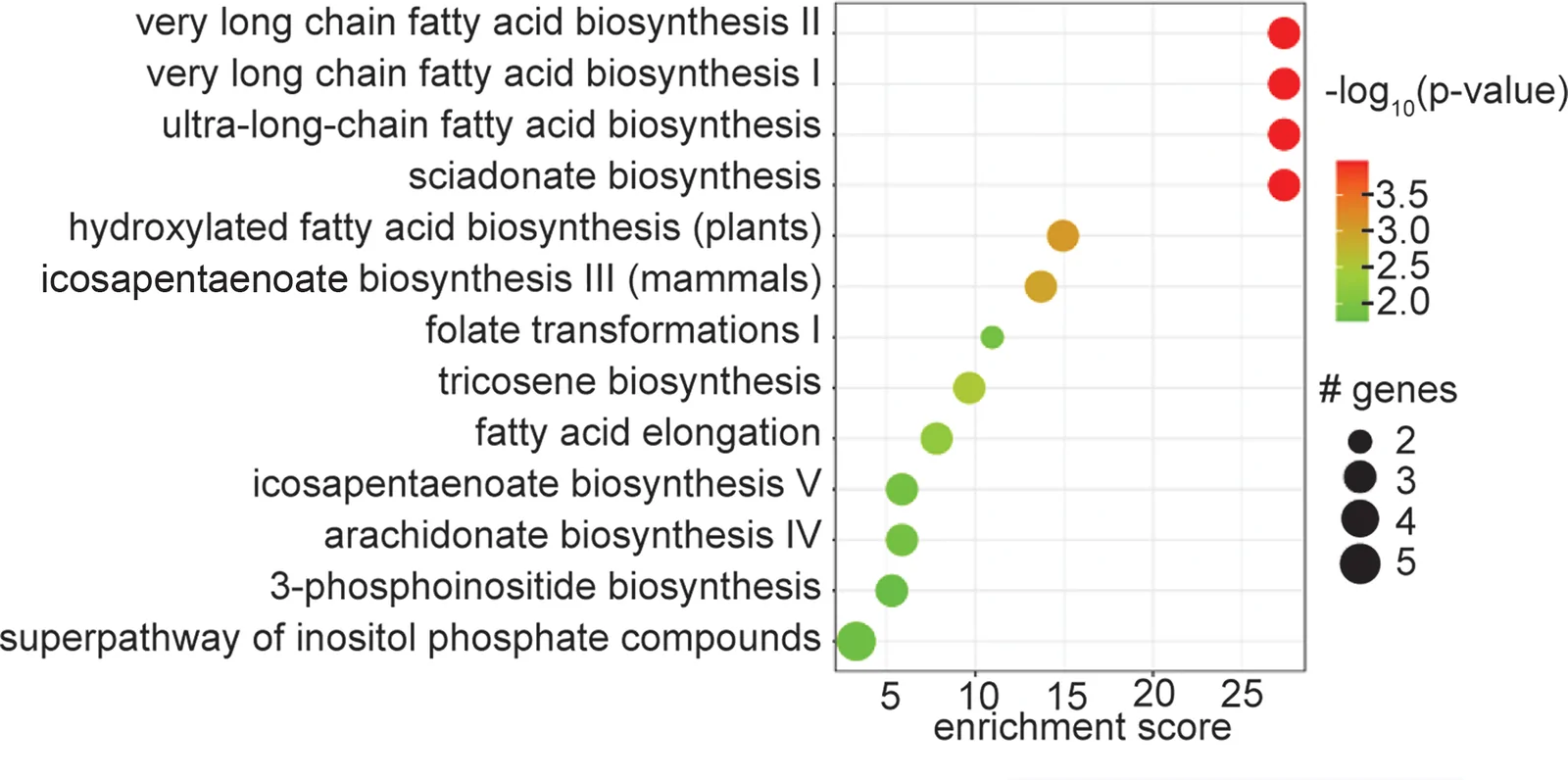
Knockout mutants sensitive to chlorpromazine reveal enriched pathways linked to lipid biosynthesis and degradation. GO analysis identifies pathways and processes enriched among genes whose knockout mutants are sensitive to chlorpromazine relative to wild-type cells.

We found 112 knockout mutants that showed sensitivity to chlorpromazine (Table 5). A metabolic enrichment pathway analysis using FungiDB (71) revealed significantly enriched pathways in fatty acid biosynthesis (GO: 0006633) and phosphatidylinositol (GO: 0006661) biosynthesis and degradation (*P*-value < 0.05) (Fig. 3).

**TABLE 5:** List of gene knockout mutants sensitive to chlorpromazine

Gene ID	Gene name	Product description
CNAG_00156	*SP1*	Putative zinc finger transcription factor
CNAG_00171		Peroxin-2
CNAG_00293	*RAS1*	Ras-like protein
CNAG_00361	*RVS167*	Hypothetical protein
CNAG_00458		Hypothetical protein, hypothetical protein, variant
CNAG_00556	*CCK1*	Casein kinase I
CNAG_00570	*PKR1*	cAMP-dependent protein kinase regulator
CNAG_00636	*CDC7*	CDC7 protein kinase
CNAG_00662		Carboxymethylenebutenolidase
CNAG_00665	*PAT1*	DNA topoisomerase 2-associated protein PAT1
CNAG_00697	*UGE1*	UDP-glucose 4-epimerase
CNAG_00715		Hypothetical protein
CNAG_00740	*SNF5*	swi/snf chromatin-remodeling complex subunit
CNAG_00760		Methylenetetrahydrofolate reductase
CNAG_01019	*SOD1*	Superoxide dismutase (Cu-Zn)
CNAG_01038		Hypothetical protein
CNAG_01040		Carboxypeptidase D
CNAG_01054	*SSU72*	RNA polymerase II subunit A domain phosphatase ssu72
CNAG_01294	*IPK1*	Inositol-pentakisphosphate 2-kinase, variant, inositol-pentakisphosphate 2-kinase
CNAG_01344		Hypothetical protein
CNAG_01381		Hypothetical protein
CNAG_01415		Cytoplasmic protein
CNAG_01419		Hypothetical protein
CNAG_01452	*MAT3*	Pheromone repeat protein
CNAG_01507	*CGI121*	tRNA modification
CNAG_01510		Hypothetical protein
CNAG_01523	*HOG1*	Mitogen-activated protein kinase
CNAG_01536		Myosin heavy chain
CNAG_01577		Glutamate dehydrogenase (NADP)
CNAG_01647	*SEC66*	Translocation protein
CNAG_01649		Hypothetical protein
CNAG_01744	*HAD1*	Phosphatase
CNAG_01837	*VPS35*	Vacuolar protein sorting-associated protein
CNAG_02029	*WSP1*	Wiskott-Aldrich syndrome protein
CNAG_02067		Vacuolar protein
CNAG_02095		G2/M cyclin
CNAG_02102		Hypothetical protein
CNAG_02172		Ubiquitin-protein ligase
CNAG_02264		AFG1-family ATPase, variant, AFG1-family ATPase
CNAG_02292		Copper chaperone
CNAG_02326	*VMA1*	V-type proton ATPase catalytic subunit A
CNAG_02357	*MKK2*	STE/STE7/MKK protein kinase
CNAG_02358		MRP family ATP-binding protein
CNAG_02473		Endoplasmic reticulum protein
CNAG_02487	*PHS1*	3-Hydroxy acyl-CoA dehydratase
CNAG_02680	*VPS15*	Protein kinase
CNAG_02687		Hypothetical protein
CNAG_02753	*LIV13*	Endoplasmic reticulum protein
CNAG_02830	*ERG4*	Delta24(24 (1))-sterol reductase
CNAG_02964		Hypothetical protein
CNAG_02975		Hypothetical protein
CNAG_03003	*SFH1*	Chromatin structure-remodeling complex subunit
CNAG_03065	*ERV14*	ER-derived vesicles protein
CNAG_03079	*PER1*	Protein processing in the ER protein 1, putative
CNAG_03080	*ELO3*	Fatty acid elongase
CNAG_03190	*CCR4*	CCR4-NOT transcription complex subunit
CNAG_03199		FAD-dependent oxidoreductase
CNAG_03206		Endonuclease III
CNAG_03370	*NCS1*	Calcium-binding protein that recruits Pik1 to Golgi membranes
CNAG_03678		Hypothetical protein
CNAG_03697		Hormone-sensitive lipase
CNAG_03821	*VPS34*	Phosphatidylinositol 3-kinase
CNAG_03834	*SUR2*	Sphinganine C4-hydroxylase involved in sphingolipid biosynthesis
CNAG_03918	*SOG2*	ram signaling network protein
CNAG_04002	*RVS161*	Hypothetical protein
CNAG_04048		brg1-associated factor b
CNAG_04113	*PTP3*	Hypothetical protein, putative protein phosphatase
CNAG_04122		Galactose dehydrogenase
CNAG_04174	*PEX6*	Peroxisomal ATPase
CNAG_04186		Hypothetical protein
CNAG_04191	*CEX1*	SCYL protein kinase
CNAG_04224	*PSR1*	Carboxy-terminal domain RNA polymerase II polypeptide A small phosphatase
CNAG_04241		Putative cytoplasmic protein
CNAG_04266	*YAF9*	YEATS domain-containing protein 4
CNAG_04316	*UTR1*	NAD + kinase
CNAG_04349		Hypothetical protein
CNAG_04456		Hypothetical protein containing phospholipid/glycerol acyltransferase domain
CNAG_04488		3-Ketoacyl-CoA reductase
CNAG_04495		Hypothetical protein
CNAG_04514	*MPK1*	CMGC/MAPK protein kinase
CNAG_04545	*CTK3*	CTD kinase subunit gamma, CTD kinase subunit gamma, variant
CNAG_04647		Glutathione synthetase
CNAG_04678	*YPK1*	AGC/AKT protein kinase
CNAG_04716		KH domain-containing putative RNA-binding protein
CNAG_04755	*BCK1*	STE/STE11/BCK1 protein kinase
CNAG_04796	*CNA1*	Calcineurin catalytic subunit
CNAG_04800		Phenylalanine-tRNA ligase, alpha subunit
CNAG_04980	*LSM1*	U6 snRNA-associated Sm-like protein
CNAG_04992		Hypothetical protein
CNAG_05222	*NRG1*	Transcriptional regulator Nrg1
CNAG_05277		Vesicle-associated membrane protein 4, vesicle-associated membrane protein 4, variant
CNAG_05573		Cytochrome c oxidase assembly protein subunit 17
CNAG_05673		GPI inositol-deacylase
CNAG_05703	*LRG1*	rho GTPase activator
CNAG_05788	*VMA10*	V-type ATPase, G subunit
CNAG_05791	*ZDS3*	zds-like protein
CNAG_05843		Translation initiation factor 4E
CNAG_06068		Chromatin structure-remodeling complex subunit RSC1/2
CNAG_06092	*CLN1*	Cyclin
CNAG_06283	*LIV4*	Putative myb-like mRNA polymerase I termination factor
CNAG_06295		Putative cytoplasmic protein
CNAG_06469	*APT1*	Phospholipid-translocating ATPase
CNAG_06645	*MTD1*	Methylenetetrahydrofolate dehydrogenase (NAD)
CNAG_06680		COP9 signalosome complex subunit 6
CNAG_06730	*GSK3*	CMGC/GSK protein kinase
CNAG_06834	*PMT1*	Dolichyl-phosphate-mannose-protein mannosyltransferase
CNAG_06997		tRNA-dihydrouridine synthase 3
CNAG_07534	*TRS130*	Trafficking protein particle complex II-specific subunit 130
CNAG_07639		Triacylglycerol lipase
CNAG_07667	*SAT4*	HAL protein kinase
CNAG_07744	*PIK1*	1-phosphatidylinositol 4-kinase
CNAG_07873		Mannosylinositol phosphoceramide (MIPC) synthase

Fatty acid synthesis is essential in *C. neoformans* (72), and fatty acids can likely be acquired from the host during growth in macrophages and during infection (73–75). An important step in fatty acid regulation is the formation of lipid droplets, where lipid species are stored and gradually released according to cellular demands for energy production, membrane synthesis, and lipid signaling (76, 77). Lipid droplets vary in size, from small droplets at the limit of light microscopy resolution (0.2–0.5 µm) to large, prominent droplets (1 µm–2.5 µm) (78).

**Fig 4:**
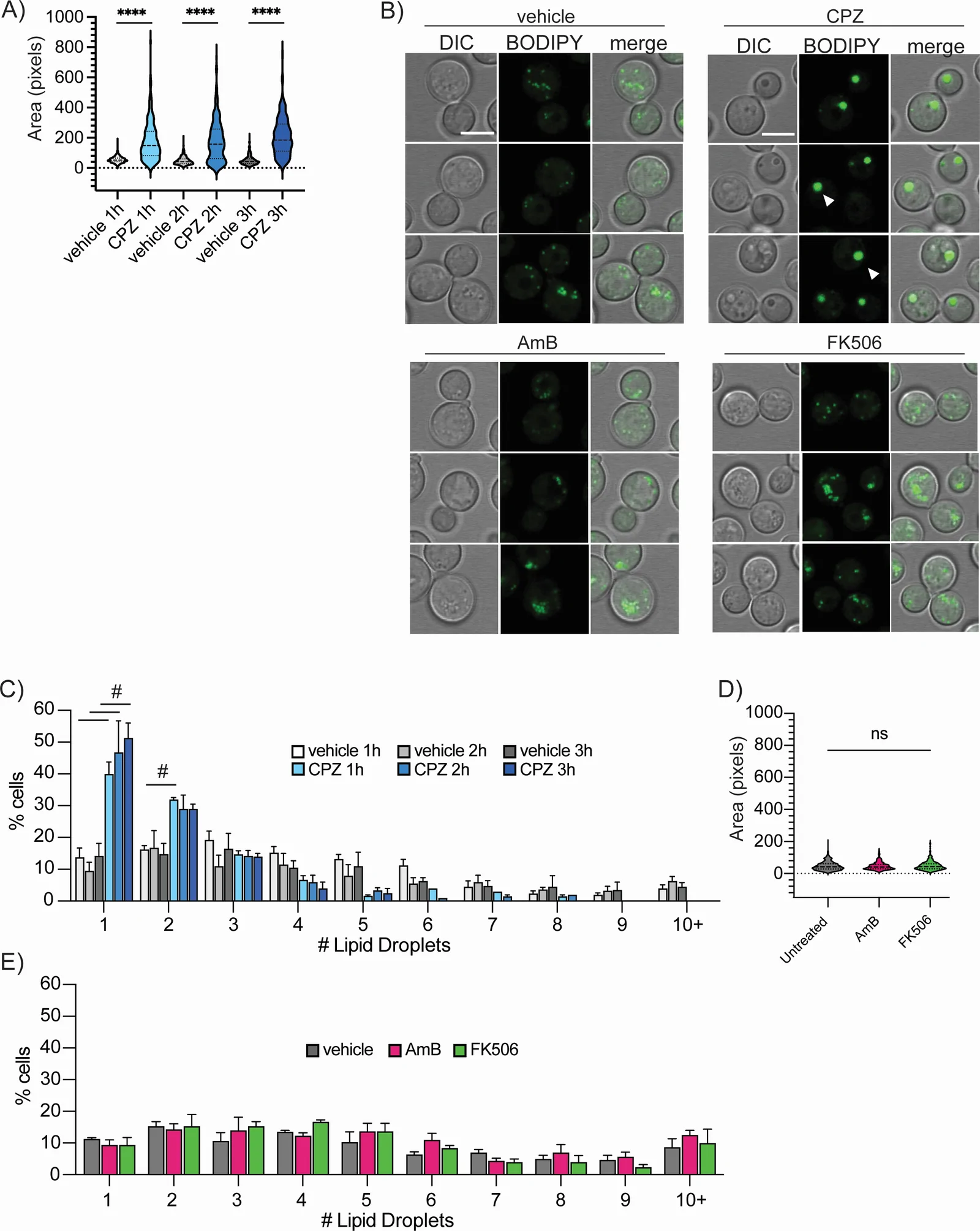
Chlorpromazine induces lipid droplet enlargement and consolidation in *C. neoformans*. (**A**) Measurement of the area of lipid droplets after 1 h, 2 h, and 3 h of incubation with chlorpromazine (100 µg/mL). Area was quantified in relative pixel size. The 25th, 50th, and 75th percentiles for area are marked as horizontal lines. (**B**) Representative confocal microscopy images of cells treated with DMSO control, chlorpromazine (100 µg/mL), amphotericin B (100 ng/mL), or FK506 (400 ng/mL) stained with BODIPY. White arrows mark enlarged lipid droplets. Scale bar: 5 µm. (**C**) Percentage of cells with a determined number of lipid droplets. The *X*-axis shows the total number of lipid droplets per cell (1–20). (**D**) Area measurement of lipid droplets from *C. neoformans* cells treated for 3 h with AmB or calcineurin inhibitor FK506. The 25th, 50th, and 75th percentiles for area are marked as vertical lines. (**E**) Confocal microscopy images of cells treated with corresponding drug and stained with BODIPY. Scale bar: 5 µm. Quantification of the number of lipid droplets per cell. We counted >100 cells per experiment and performed three independent experiments. Statistical significance was determined using Mann-Whitney (parts **A and D**, ****P < 0.0001) or unpaired *t*-test (parts **C and E**, # *P* < 0.05.

To assess the effect of chlorpromazine on lipid droplet formation, we used the fluorescent probe BODIPY 493/503, which stains neutral lipids (79) and is widely used to visualize and measure lipid droplet size and number in fungal cells (80). We found that lipid droplet area significantly increased with chlorpromazine (Fig. 4A). This increase was time-dependent, as lipid droplet area significantly increased when cells were treated for 3 h compared to 1 h (*P*-value: 0.0037; Mann-Whitney), suggesting a dynamic lipid droplet change over time with drug treatment. Quantification of the number of lipid droplets per cell showed that chlorpromazine-treated cells also had fewer lipid droplets, with 30%–50% of cells containing only one or two lipid droplets per cell (Fig. 4B). We noticed that CPZ is sometimes visible around lipid droplets (Fig. S3). It is possible that the formation of enlarged lipid droplets may help sequester chlorpromazine, potentially containing the drug and preventing further intracellular damage. DMSO (vehicle)-treated cells had a larger distribution in the number of lipid droplets, ranging from 1 to 20 lipid droplets per cell (Fig. 4C). These results suggest that chlorpromazine reduces the number of small lipid droplets to favor the formation of enlarged lipid droplets in *C. neoformans*. The disproportionate enlargement of lipid droplets can have both beneficial and detrimental effects. Although the enlargement of lipid droplets can still provide storage capacity for excess lipids and protect from lipotoxicity, persistent lipid accumulation disrupts signaling pathways, membrane trafficking, and protein synthesis (81–84).

To verify that the formation of enlarged lipid droplets was specific to chlorpromazine, we treated fungal cells with 400 ng/mL of the calcineurin inhibitor FK506 (85) or 100 ng/mL AmB (Fig. 4B). AmB and FK506 are not known to have a direct effect on other fatty acids. Neither drug increased lipid droplet area (Fig. 4D) or number compared with the control (Fig. 4E). These results show that stress unrelated to lipid synthesis does not induce changes to lipid droplet number or size, and that the effect is specific to chlorpromazine.

We then selected a subset of eight lipid biosynthesis gene mutants that showed the greatest chlorpromazine sensitivity. The BODIPY staining was lower in mutants than in wild-type cells and still increased with CPZ treatment, but mutants exhibited a complete inability or decreased ability (*cnag_03080*Δ or *cnag_04488*Δ) to form one to two enlarged, consolidated lipid droplets per cell when treated with CPZ (Fig. S4). These data are consistent with the hypothesis that chlorpromazine increases lipid sequestration in enlarged droplets and demonstrate that lipid droplet formation correlates with CPZ resistance.

Our screen also identified eight knockout mutants resistant to chlorpromazine belonging to the vacuolar protein sorting process and part of the ESCRT pathway: *vps20*Δ, *vps22*Δ*, vps23*Δ, *vps25*Δ*, vps27*Δ*, vps28*Δ*, vps32*Δ*,* and *vps36*Δ (Fig. S5). ESCRT is involved in multivesicular body formation, membrane remodeling, and membrane protein degradation (86–89). However, ESCRT mutants in *S. cerevisiae* cannot break down lipid droplets (90), suggesting a connection between lipid droplets and ESCRT function that merits additional study.

### Chlorpromazine analogs act synergistically with fluconazole and AmB

**Fig 5:**
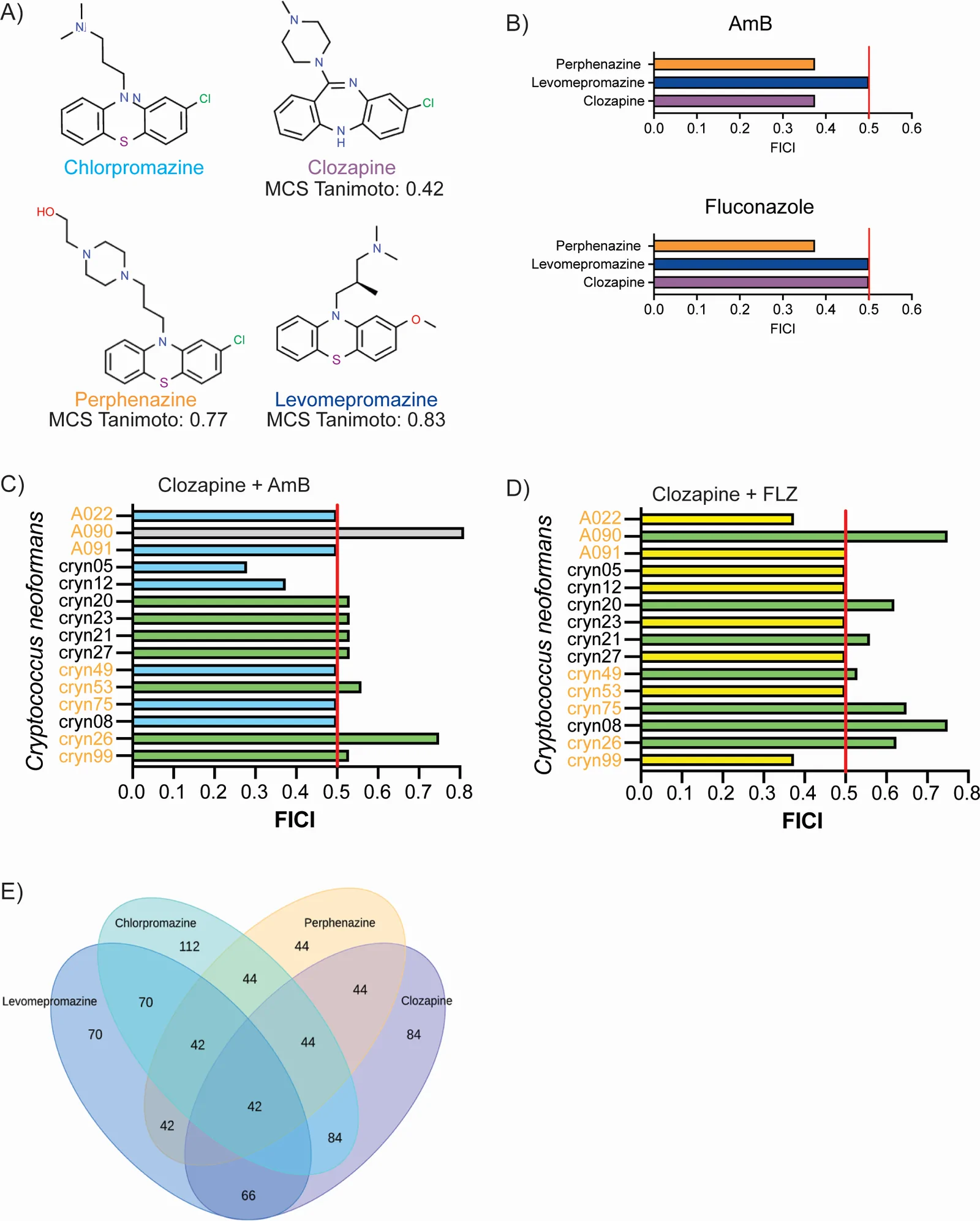
Chlorpromazine analogs act synergistically with AmB and fluconazole. (**A**) Chlorpromazine analogs, clozapine, perphenazine, and levomepromazine, with different degrees of structural similarity. (**B**) Synergistic FICI scores of the different analogs in combination with AmB (Top) or fluconazole (bottom). FICI scores of clozapine in combination with (**C**) AmB and (**D**) fluconazole. Blue (AmB) or yellow (fluconazole) bars represent an FICI score of ≤0.5, indicating a synergistic result. Gray bars represent an indifferent interaction, and green bars represent an additive interaction. The red vertical line marks the division between synergistic and non-synergistic at the FICI = 0.5 mark. Names of strains resistant to fluconazole are labeled in orange. (**E**) Venn diagram showing the overlap of single-gene knockout mutants sensitive to chlorpromazine and its three analogs. The numbers below each analog represent the subset of mutants that were sensitive to the respective drug among the mutants sensitive to chlorpromazine. Chlorpromazine-sensitive mutants were identified from an entire single-gene knockout mutant library screen.

While we have previously tested synergistic combinations in mouse infection models (24), chlorpromazine treatment in rodents presents several challenges that made finding an appropriate dose that did not cause harm difficult. Specifically, chlorpromazine affects cellular immune responses (91, 92), causing agranulocytosis (93), loss of alveolar macrophages in rats (94), and suppression of IL-6 in monocytes. We therefore decided to test small molecules with chemical structures similar to chlorpromazine that could have similar effects against *C. neoformans* while being safer for the mammalian hosts. Using ChemMine tools’ Maximum Common Substructure (MCS) Tanimoto score (95) to calculate similarity, we found three different chlorpromazine analogs with varying chemical substructure similarity: clozapine, perphenazine, and levomepromazine, with MCS Tanimoto scores of 0.42, 0.77, and 0.83, respectively (Fig. 5A). Clozapine is less likely to cause motor side effects (96) and perphenazine (97) and levomepromazine (98) exhibit lower incidence of immunomodulatory side effects. Perphenazine and levomepromazine are first-generation antipsychotics. Clozapine is a second-generation antipsychotic known for its higher effectiveness and reduced adverse side effects compared to other atypical antipsychotics and chlorpromazine (99, 100). When tested for synergy with AmB and fluconazole against our lab strain KN99, all three chlorpromazine analogs acted synergistically with both antifungals regardless of structural similarity (Fig. 5B). We further tested clozapine in combination with AmB or fluconazole against *C. neoformans* clinical isolates and both combinations, respectively, showed synergy in 46% and 53% of the isolates tested (Fig. 5C and D). When the drug combination did not induce a synergistic response from an isolate, the response was almost always additive (13 of 14 isolates) and indifferent in only one isolate. We did not observe an antagonistic response to these drug combinations from any isolate.

We tested the growth of mutants resistant to chlorpromazine in the presence of chlorpromazine analogs and found that resistance to CPZ conferred resistance to the analogs (data not shown). We further investigated whether the sensitive knockout mutants were affected by clozapine, levomepromazine, and perphenazine (Tables 6 to 8). Only 44 of the 112 knockout mutants sensitive to chlorpromazine were also sensitive to perphenazine. In comparison, clozapine and levomepromazine induced sensitivity in 84% and 70% of the knockout mutants, respectively (Fig. 5E). The genes whose knockout mutants were sensitive to clozapine, levomepromazine, and perphenazine were, like genes whose knockout mutants were sensitive to CPZ, enriched for genes involved in the fatty acid biosynthetic process (GO: 0006633) (71). These results show these analogs can still target the same biological functions as chlorpromazine and synergize with both AmB and fluconazole, demonstrating their potential for safer antifungal combination therapy.

**TABLE 6:** List of gene knockout mutants sensitive to clozapine

Gene ID	Gene name	Product description
CNAG_00156	*SP1*	Putative zinc finger transcription factor
CNAG_00171		Peroxin-2
CNAG_00293	*RAS1*	Ras-like protein
CNAG_00361	*RVS167*	Hypothetical protein RVS167
CNAG_00556	*CCK1*	Casein kinase I
CNAG_00570	*PKR1*	cAMP-dependent protein kinase regulator
CNAG_00636	*CDC7*	CDC7 protein kinase
CNAG_00662		Carboxymethylenebutenolidase
CNAG_00665	*PAT1*	DNA topoisomerase 2-associated protein PAT1
CNAG_00697	*UGE1*	UDP-glucose 4-epimerase
CNAG_00740	*SNF5*	SWI/SNF chromatin-remodeling complex subunit
CNAG_00760		Methylenetetrahydrofolate reductase
CNAG_01019	*SOD1*	Superoxide dismutase (Cu-Zn)
CNAG_01040		Carboxypeptidase D
CNAG_01054	*SSU72*	RNA polymerase II subunit A domain phosphatase ssu72
CNAG_01294	*IPK1*	Inositol-pentakisphosphate 2-kinase, variant, inositol-pentakisphosphate 2-kinase
CNAG_01415		Cytoplasmic protein
CNAG_01452	*MAT3*	Pheromone repeat protein
CNAG_01507	*CGI121*	tRNA modification
CNAG_01523	*HOG1*	Mitogen-activated protein kinase
CNAG_01536		Myosin heavy chain
CNAG_01577		Glutamate dehydrogenase (NADP)
CNAG_01647	*SEC66*	Translocation protein
CNAG_01837	*VPS35*	Vacuolar protein sorting-associated protein
CNAG_02029	*WSP1*	Wiskott-Aldrich syndrome protein
CNAG_02264		AFG1-family ATPase, variant, AFG1-family ATPase
CNAG_02292		Copper chaperone
CNAG_02326	*VMA1*	V-type proton ATPase catalytic subunit A
CNAG_02358		MRP family ATP-binding protein
CNAG_02473		Endoplasmic reticulum protein
CNAG_02487	*PHS1*	3-hydroxy acyl-CoA dehydratase
CNAG_02680	*VPS15*	Protein kinase
CNAG_02753	*LIV13*	Endoplasmic reticulum protein
CNAG_02830	*ERG4*	Delta24(24 (1))-sterol reductase
CNAG_03003	*SFH1*	Chromatin structure-remodeling complex subunit
CNAG_03065	*ERV14*	ER-derived vesicles protein
CNAG_03079	*PER1*	Protein processing in the ER protein 1, putative
CNAG_03080	*ELO3*	Fatty acid elongase
CNAG_03190	*CCR4*	CCR4-NOT transcription complex subunit
CNAG_03199		FAD-dependent oxidoreductase
CNAG_03206		Endonuclease III
CNAG_03370	*NCS1*	Calcium-binding protein that recruits Pik1 to Golgi membranes
CNAG_03697		Hormone-sensitive lipase
CNAG_03821	*VPS34*	Phosphatidylinositol 3-kinase
CNAG_03834	*SUR2*	Sphinganine C4-hydroxylase involved in sphingolipid biosynthesis
CNAG_03918	*SOG2*	ram signaling network protein
CNAG_04048		brg1-associated factor b
CNAG_04122		Galactose dehydrogenase
CNAG_04191	*CEX1*	SCYL protein kinase
CNAG_04224	*PSR1*	Carboxy-terminal domain RNA polymerase II polypeptide A small phosphatase
CNAG_04266	*YAF9*	YEATS domain-containing protein 4
CNAG_04316	*UTR1*	NAD + kinase
CNAG_04488		3-Ketoacyl-CoA reductase
CNAG_04514	*MPK1*	CMGC/MAPK protein kinase
CNAG_04545	*CTK3*	CTD kinase subunit gamma, CTD kinase subunit gamma, variant
CNAG_04647		Glutathione synthetase
CNAG_04678	*YPK1*	AGC/AKT protein kinase
CNAG_04716		KH domain-containing putative RNA-binding protein
CNAG_04755	*BCK1*	STE/STE11/BCK1 protein kinase
CNAG_04796	*CNA1*	Calcineurin catalytic subunit
CNAG_04800		Phenylalanine-tRNA ligase, alpha subunit
CNAG_04980	*LSM1*	U6 snRNA-associated Sm-like protein
CNAG_05222	*NRG1*	Transcriptional regulator Nrg1
CNAG_05277		Vesicle-associated membrane protein 4, vesicle-associated membrane protein 4, variant
CNAG_05673		GPI inositol-deacylase
CNAG_05703	*LRG1*	rho GTPase activator
CNAG_05788	*VMA10*	V-type ATPase, G subunit
CNAG_05791	*ZDS3*	zds-like protein
CNAG_05843		Translation initiation factor 4E
CNAG_06068		Chromatin structure-remodeling complex subunit RSC1/2
CNAG_06092	*CLN1*	Cyclin
CNAG_06283	*LIV4*	Putative myb-like mRNA polymerase I termination factor
CNAG_06295		Putative cytoplasmic protein
CNAG_06469	*APT1*	Phospholipid-translocating ATPase
CNAG_06645	*MTD1*	Methylenetetrahydrofolate dehydrogenase (NAD)
CNAG_06680		COP9 signalosome complex subunit 6
CNAG_06730	*GSK3*	CMGC/GSK protein kinase
CNAG_06834	*PMT1*	Dolichyl-phosphate-mannose-protein mannosyltransferase
CNAG_06997		tRNA-dihydrouridine synthase 3
CNAG_07534	*TRS130*	Trafficking protein particle complex II-specific subunit 130
CNAG_07639		Triacylglycerol lipase
CNAG_07667	*SAT4*	HAL protein kinase
CNAG_07744	*PIK1*	1-Phosphatidylinositol 4-kinase
CNAG_07873		Mannosylinositol phosphoceramide (MIPC) synthase

**TABLE 7:** List of gene knockout mutants sensitive to levomepromazine

Gene ID	Gene name	Product description
CNAG_00156	*SP1*	Putative zinc finger transcription factor
CNAG_00171		Peroxin-2
CNAG_00293	*RAS1*	Ras-like protein
CNAG_00361	*RVS167*	Hypothetical protein
CNAG_00570	*PKR1*	cAMP-dependent protein kinase regulator
CNAG_00636	*CDC7*	CDC7 protein kinase
CNAG_00662		Carboxymethylenebutenolidase
CNAG_00665	*PAT1*	DNA topoisomerase 2-associated protein PAT1
CNAG_00697	*UGE1*	UDP-glucose 4-epimerase
CNAG_00740	*SNF5*	SWI/SNF chromatin-remodeling complex subunit
CNAG_00760		Methylenetetrahydrofolate reductase
CNAG_01040		Carboxypeptidase D
CNAG_01054	*SSU72*	RNA polymerase II subunit A domain phosphatase ssu72
CNAG_01415		Cytoplasmic protein
CNAG_01523	*HOG1*	Mitogen-activated protein kinase
CNAG_01536		Myosin heavy chain
CNAG_01577		Glutamate dehydrogenase (NADP)
CNAG_01647	*SEC66*	Translocation protein
CNAG_01837	*VPS35*	Vacuolar protein sorting-associated protein
CNAG_02029	*WSP1*	Wiskott-Aldrich syndrome protein
CNAG_02067		Vacuolar protein
CNAG_02292		Copper chaperone
CNAG_02358		MRP family ATP-binding protein
CNAG_02473		Endoplasmic reticulum protein
CNAG_02487	*PHS1*	3-Hydroxy acyl-CoA dehydratase
CNAG_02680	*VPS15*	Protein kinase
CNAG_03003	*SFH1*	Chromatin structure-remodeling complex subunit
CNAG_03065	*ERV14*	ER-derived vesicles protein
CNAG_03079	*PER1*	Protein processing in the ER protein 1, putative
CNAG_03080	*ELO3*	Fatty acid elongase
CNAG_03190	*CCR4*	CCR4-NOT transcription complex subunit
CNAG_03206		Endonuclease III
CNAG_03697		Hormone-sensitive lipase
CNAG_03821	*VPS34*	Phosphatidylinositol 3-kinase
CNAG_03834	*SUR2*	Sphinganine C4-hydroxylase involved in sphingolipid biosynthesis
CNAG_03918	*SOG2*	ram signaling network protein
CNAG_04048		brg1-associated factor b
CNAG_04174	*PEX6*	Peroxisomal ATPase
CNAG_04191	*CEX1*	SCYL protein kinase
CNAG_04224	*PSR1*	Carboxy-terminal domain RNA polymerase II polypeptide A small phosphatase
CNAG_04241		Putative cytoplasmic protein
CNAG_04266	*YAF9*	YEATS domain-containing protein 4
CNAG_04316	*UTR1*	NAD + kinase
CNAG_04488		3-Ketoacyl-CoA reductase
CNAG_04514	*MPK1*	CMGC/MAPK protein kinase
CNAG_04545	*CTK3*	CTD kinase subunit gamma, CTD kinase subunit gamma, variant
CNAG_04647		Glutathione synthetase
CNAG_04716		KH domain-containing putative RNA-binding protein
CNAG_04755	*BCK1*	STE/STE11/BCK1 protein kinase
CNAG_04796	*CNA1*	Calcineurin catalytic subunit
CNAG_04800		Phenylalanine-tRNA ligase, alpha subunit
CNAG_04980	*LSM1*	U6 snRNA-associated Sm-like protein
CNAG_05277		Vesicle-associated membrane protein 4, vesicle-associated membrane protein 4, variant
CNAG_05573		Cytochrome c oxidase assembly protein subunit 17
CNAG_05673		GPI inositol-deacylase
CNAG_05703	*LRG1*	rho GTPase activator
CNAG_05791	*ZDS3*	zds-like protein
CNAG_05843		Translation initiation factor 4E
CNAG_06068		Chromatin structure-remodeling complex subunit RSC1/2
CNAG_06092	*CLN1*	Cyclin
CNAG_06469	*APT1*	Phospholipid-translocating ATPase
CNAG_06645	*MTD1*	Methylenetetrahydrofolate dehydrogenase (NAD)
CNAG_06680		COP9 signalosome complex subunit 6
CNAG_06730	*GSK3*	CMGC/GSK protein kinase
CNAG_06834	*PMT1*	Dolichyl-phosphate-mannose-protein mannosyltransferase
CNAG_06997		tRNA-dihydrouridine synthase 3
CNAG_07534	*TRS130*	Trafficking protein particle complex II-specific subunit 130
CNAG_07667	*SAT4*	HAL protein kinase
CNAG_07744	*PIK1*	1-Phosphatidylinositol 4-kinase
CNAG_07873		Mannosylinositol phosphoceramide (MIPC) synthase

**TABLE 8:** List of gene knockout mutants sensitive to perphenazine

Gene ID	Gene name	Product description
CNAG_00156	*SP1*	Putative zinc finger transcription factor
CNAG_00361	*RVS167*	Hypothetical protein
CNAG_00636	*CDC7*	CDC7 protein kinase
CNAG_00662		Carboxymethylenebutenolidase
CNAG_00665	*PAT1*	DNA topoisomerase 2-associated protein PAT1
CNAG_00697	*UGE1*	UDP-glucose 4-epimerase
CNAG_00740	*SNF5*	SWI/SNF chromatin-remodeling complex subunit
CNAG_00760		Methylenetetrahydrofolate reductase
CNAG_01523	*HOG1*	Mitogen-activated protein kinase
CNAG_01536		Myosin heavy chain
CNAG_01577		Glutamate dehydrogenase (NADP)
CNAG_01647	*SEC66*	Translocation protein
CNAG_02292		Copper chaperone
CNAG_02487	*PHS1*	3-Hydroxy acyl-CoA dehydratase
CNAG_02680	*VPS15*	Protein kinase
CNAG_02830	*ERG4*	Delta24(24(1))-sterol reductase
CNAG_03003	*SFH1*	Chromatin structure-remodeling complex subunit
CNAG_03065	*ERV14*	ER-derived vesicles protein
CNAG_03080	*ELO3*	Fatty acid elongase
CNAG_03190	*CCR4*	CCR4-NOT transcription complex subunit
CNAG_03697		Hormone-sensitive lipase
CNAG_03821	*VPS34*	Phosphatidylinositol 3-kinase
CNAG_03918	*SOG2*	ram signaling network protein
CNAG_04048		53 kDa brg1-associated factor b
CNAG_04266	*YAF9*	YEATS domain-containing protein 4
CNAG_04316	*UTR1*	NAD kinase
CNAG_04488		3-Ketoacyl-CoA reductase
CNAG_04514	*MPK1*	CMGC/MAPK protein kinase
CNAG_04545	*CTK3*	CTD kinase subunit gamma
CNAG_04647		Glutathione synthetase
CNAG_04716		KH domain-containing protein
CNAG_04755	*BCK1*	STE/STE11/BCK1 protein kinase
CNAG_04796	*CNA1*	Calcineurin a catalytic subunit
CNAG_05222	*NRG1*	Transcriptional regulator Nrg1
CNAG_05791	*ZDS3*	zds-like protein
CNAG_06068		Chromatin structure-remodeling complex subunit RSC1/2
CNAG_06092	*CLN1*	Cyclin
CNAG_06469	*APT1*	Phospholipid-translocating ATPase APT1
CNAG_06730	*GSK3*	CMGC/GSK protein kinase
CNAG_06834	*PMT1*	Dolichyl-phosphate-mannose-protein mannosyltransferase
CNAG_07534	*TRS130*	Trafficking protein particle complex II-specific subunit 130
CNAG_07667	*SAT4*	HAL protein kinase
CNAG_07744	*PIK1*	1-Phosphatidylinositol 4-kinase
CNAG_07873		MIPC synthase

## DISCUSSION

Synergistic drug combinations are promising potential treatments for systemic fungal infections. They can overcome resistance (Fig. 1), lower treatment costs, and delay the development of drug resistance (30, 101). This study aims to elucidate the molecular mechanisms underlying the synergistic interactions between AmB and the antipsychotic chlorpromazine, revealing a potent combination with enhanced antifungal efficacy. Moreover, chlorpromazine’s less toxic analogs, perphenazine, levomepromazine, and clozapine, appear to act via the same mechanism and are themselves promising antifungal treatments.

**Fig 6:**
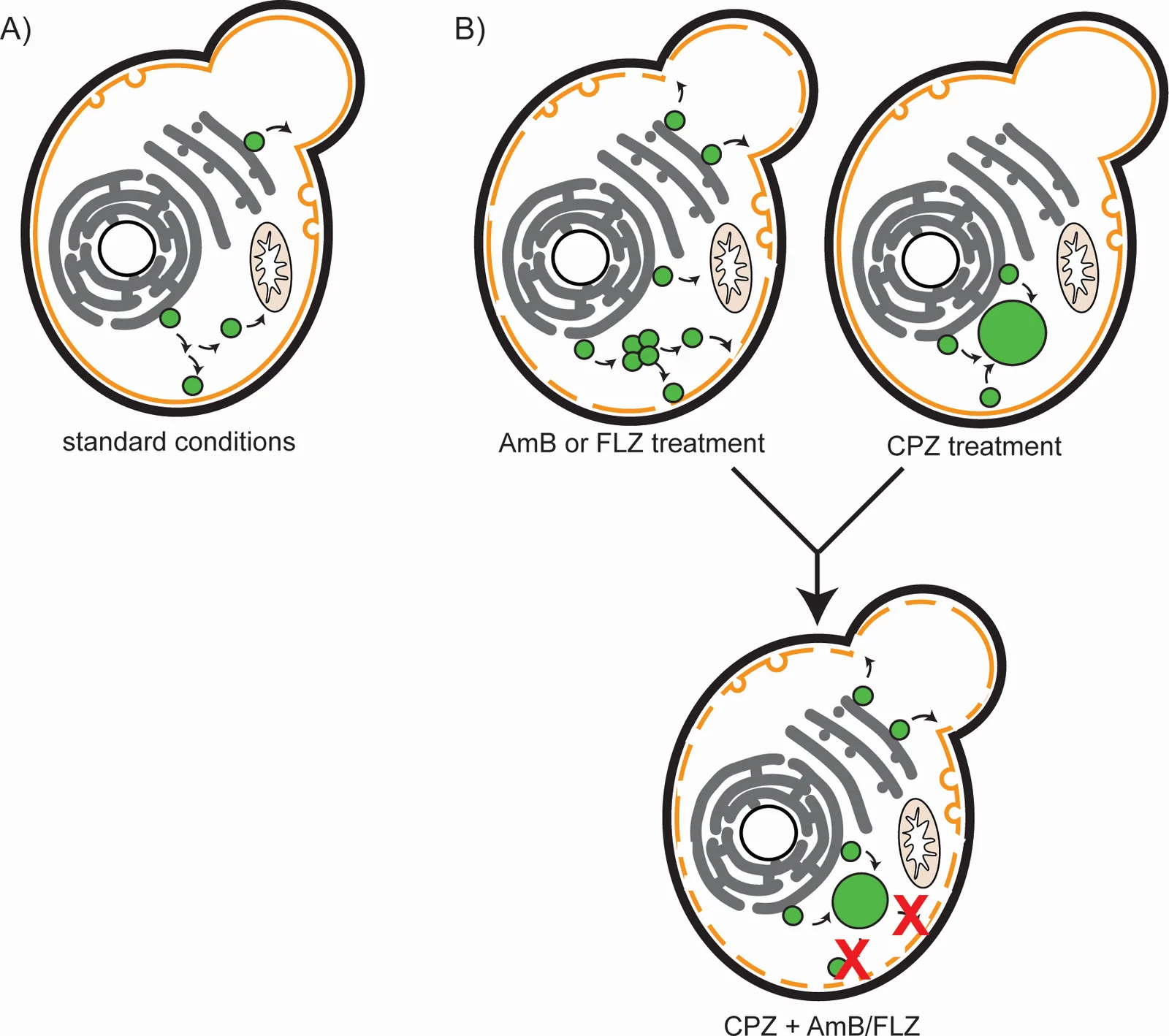
Chlorpromazine + AmB act together to damage fungal cells. (**A**) Under standard growth conditions, lipid droplets (green circles) traffic between the ER and mitochondria or the plasma membrane or the Golgi and plasma membrane. (**B**) Treatment with chlorpromazine increases lipid droplet size, potentially leading to increased lipid sequestration and slowing the cellular response to sterol stress. Since chlorpromazine and AmB together increase membrane permeability and cell death, the bioavailability of the two drugs increases as exposure continues, accelerating lipid droplet formation and decreasing the ability of fungal cells to adapt to these drugs.

Previous research showed that chlorpromazine could interact with cellular membranes in different organisms and cell types (102). The effects of this disruption extend from membrane dissolution in very high concentrations to increased membrane permeability with lower drug concentrations. Others showed that CPZ has a reduced affinity for membranes that contain higher cholesterol levels, the analog of ergosterol in mammalian cells (103). Here, we saw that CPZ increases lipid droplet size, a response to stress that phenocopies nutrient starvation (76) and allows regulated breakdown of lipid droplets to prevent lipotoxicity (104) in mitochondria and other organelles. In combination with AmB, chlorpromazine effectively disrupts membranes, increasing membrane permeability fivefold (Fig. 1B). However, with lipids sequestered in large lipid droplets, the cell might not be able to repair the plasma membrane, resulting in severe cell damage (Fig. 6). This is consistent with results from *S. cerevisiae*, which show that lipid droplet formation is induced by lipid stress (105). Another, not mutually exclusive, hypothesis is based on the fact that other phenothiazines inhibit calmodulin (106), including directly binding to *C. neoformans* calmodulin (36). Since calmodulin inhibition is part of the cellular stress response, it could be that combined inhibition of multiple stress response pathways (107), rather than lipid droplet formation specifically, results in the synergistic interaction between AmB and CPZ.

Chlorpromazine is not the only psychiatric drug with antifungal properties: the selective serotonin reuptake inhibitor sertraline also inhibits fungal cell growth (108, 109) and increases lipid droplet size in *C. neoformans* (110). The authors proposed that the increase in the size of lipid droplets was due to a surplus of phosphatidic acid, which induces lipid droplet fusion in *S. cerevisiae*. When there is an increase in the number of lipid droplets in cells, droplet fusion events increase, favoring the eventual coalescence of lipid droplets (77). We hypothesize that this occurs when chlorpromazine-treated cells show enlarged lipid droplets (Fig. 4).

Phenothiazines have attracted attention for their antifungal activity before (35, 36). Recent work created derivatives of the phenothiazine, fluphenazine, with activity against *C. neoformans* and *C. albicans* (111), and, in the case of one derivative, over a dozen species of medically relevant fungi (112). One derivative exhibited activity against fluconazole-resistant *C. albicans* (111), but the derivative also acted antagonistically with fluconazole against *C. albicans* but not *Nakaseomyces glabratus* (formerly *Candida glabrata* [113]. Fluphenazine induced the expression of ABC transporter genes (114, 115), but the derivative was not exported by these transporters (113). When combined with our data, this work demonstrates the importance of studying drug interactions in a variety of isolates and species. It also suggests that chlorpromazine might change plasma membrane composition by altering transporter protein expression, which, in turn, can decrease the bioavailability of other drugs.

Chlorpromazine analogs are promising potential antifungal drugs. We demonstrated that chlorpromazine analogs in clinical use show synergy with AmB and fluconazole against *C. neoformans* and AmB against *C. albicans*. Clozapine, a second-generation antipsychotic, has a lower risk of certain adverse effects compared to chlorpromazine. Future research can further optimize these analogs for enhanced efficacy and safety profiles against fungal infections. These results highlight the potential of repurposing antipsychotic analogs for the development of novel treatments for fungal infections.

## MATERIALS AND METHODS

### *C. neoformans* growth and small-molecule MIC determination

MIC_90_ assays were performed with 1× yeast nitrogen base (YNB) + 2% glucose. Overnight cultures were grown at 37°C and diluted to OD_600_ = 0.0292. Small molecules were dissolved in DMSO at varying stock concentrations and serially diluted in a twofold series in a 96-well plate. Each well was inoculated, and plates were incubated for 48 h at 30°C. OD_600_ was measured using the Molecular Devices SpectraMax iD5 plate reader. Growth was calculated using the formula [(OD_600_ well × at *t* = 48 h) − (OD_600_ well × at *t* = 0 h)]/(OD_600_ well no drug at *t* = 48 h). The lowest drug concentration showing growth at 0.1 (90% inhibition) was considered the MIC_90_. Resistance is defined as >64 µg/mL for fluconazole and >2 µg/mL for amphotericin B. Susceptible-dose dependent is defined as 16–32 µg/mL for fluconazole. Clinical isolates were gifted from Kimberly Hanson, M.D., ARUP Laboratories, Salt Lake City, UT, USA.

### Checkerboard assays

Cells were grown overnight in YNB and adjusted to OD_600_ = 0.03 as starting inoculum for all species of fungi. Drugs were serially diluted in 96-well plates as described previously (24), and all wells were inoculated with 2 µL of cells. Plates were incubated at 30°C with shaking/resuspension at 24 and 48 h. OD_600_ for each plate was measured at 0 and 48 h with a Molecular Devices SpectraMax iD5 plate reader for growth inhibition assessment. FICI scores were calculated as described in Wambaugh et al. (116). Briefly, FICI scores are determined by a 90% growth inhibition cutoff for MICs using this equation:


$$FICI\;=\;\left(\frac{concentration\;of\;drug\;1\;in\;well}{MIC_{90}\;of\;drug\;1}\right)+\left(\frac{concentration\;of\;drug\;2\;in\;well}{MIC_{90}\;of\;drug\;2}\right)$$

FICI scores of ≤0.5 are considered synergistic. Checkerboard assays are repeated at least twice for each strain and drug combination. If the result (synergistic or not synergistic) is the same, the average FICI is shown on graphs. If the result is not the same (e.g., one synergistic, one not), the assay is repeated a minimum of five times and the scores from the predominant result (i.e., all scores over 0.5 or all scores < 0.5) are averaged and shown on the graph.

### Cell viability assays

Cells were grown overnight and then subcultured to YNB + vehicle (DMSO) or small molecules. Cultures were then grown at 30°C with constant rotation for 3 hours, then washed twice with 1× phosphate-buffered saline (PBS). Samples were diluted in 1× PBS to yield a countable number of colonies per plate (~50–200 colonies), and 100 μL and 200 μL plated onto two different YPD plates as technical replicates. After 48 h at 30°C, colonies were counted, technical replicates were averaged, and CFUs were normalized to the vehicle control. This experiment was repeated three independent times to produce biological replicates.

### Cell permeability assays

Cells were grown overnight and then subcultured to YNB + vehicle (DMSO) or small molecules. Cultures were then grown at 30°C with constant rotation for 3 h, then washed twice with 1× PBS. We added propidium iodide to 3 μg/mL, then analyzed by flow cytometry. Voltage gates were the same as described in reference 24: FSC, 500; SSC, 310; and PE, 496. In total, 100,000 cells were counted per sample.

### RT-PCR

Quantification of ergosterol biosynthesis pathway gene expression intermediates was done by RT-PCR. *C. neoformans* cells were treated overnight with each drug. Cells (1 × 10^7^) were pelleted, flash-frozen in liquid nitrogen, and lyophilized until the pellet was completely dry. RNA was extracted using the Qiagen RNeasy column kit (Catalog no. 74014) with modifications. The pellet was resuspended in 600 µL of RTL buffer, and approximately 50 µL of 1 mm and 150 µL of 0.5 mm zirconium beads were added to each sample. Samples were placed in the BioSpec mini bead beater to lyse cells with 12 × 2 min pulsations, placing blocks and samples on ice for 5 min in between pulses to prevent overheating. RNA was then extracted following kit instructions. cDNA was synthesized using random primers (Invitrogen), M-MLV reverse transcriptase (Invitrogen), 10× dNTPs, and incubated at 37°C for 1 h. cDNA was further purified using Zymo DNA Clean & Concentrator (Cat. no. 11303) following kit instructions. Real-time qPCR was performed using the Applied Biosystems QuantStudio 3 system and SYBR Green for continuous fluorescent monitoring. *C*_*t*_ values were obtained in triplicate, averaged, and normalized to ACT1, calculating relative gene expression using the 2^−∆∆*Ct*^ method.

### Knockout mutant library drug agar screening

Drug agar plates were made by adding small molecules dissolved in DMSO into 2× YNB + 4% glucose and mixing a 50:50 ratio of drug/YNB and agar for a final concentration of 2% agar. Targeted single knockout mutant collections were constructed by Boucher et al. (117) and Lee et al. (70) and purchased from the Fungal Genetics Stock Center. Frozen stocks of the mutants were pinned onto YPAD agar to grow at 30°C, and liquid cultures were made in 1× YNB + 2% agar. Liquid cultures were pinned onto drug agar plates using a 96-well replicator pinner. Plates were then incubated at 30°C, and images were taken every day for 4–5 days using a flatbed scanner.

### Small-molecule sensitivity testing

*C. neoformans* strain WT or single-gene knockout mutants obtained from the Madhani collection (31) were streaked onto YPAD agar and grown at 30°C for at least 48 h. Liquid cultures were made in 1× YNB + 2% glucose and incubated overnight. Fungal cells were adjusted to an initial concentration of 1 × 10^7^ cells/mL and fivefold serially diluted across six wells in a 96-well plate. Three microliters of each dilution was pipetted using a multichannel pipette onto each plate containing YNB agar + drug (300 μg/mL CPZ, 450 μg/mL clozapine, 150 μg/mL perphenazine, or 270 μg/mL levomepromazine) or DMSO control. Agar plates were incubated at 30°C, and images were taken daily for 4–5 days using a flatbed scanner. The spot assay was repeated twice, and representative images are shown.

### Confocal microscopy with BODIPY staining

Liquid cultures of *C. neoformans* strain KN99 and knockout mutants were grown in YPD medium at 37°C overnight. Fungal cells were adjusted to 3 × 10^6^ cells/mL and treated with the appropriate drug for 3 h (100 μg/mL clozapine, 100 μg/mL CPZ, 100 ng/mL AmB, or 400 ng/mL FK506), then centrifuged and washed with 1× YPD. For chlorpromazine time-course experiments, cells were treated with 100 µg/mL of CPZ for 1, 2, and 3 h. Cells were then treated with BODIPY stain for 30 min. BODIPY stain was dissolved from powder in anhydrous DMSO to a 10 mM solution and adjusted to 1 mM. BODIPY solution was added to each sample to achieve a final concentration of 3 μM. After staining, the cells were washed twice with 1× PBS and resuspended in 1× PBS for microscopy.

Cells were prepared for microscopy in a MatTek glass-bottom dish. Images were taken using the Zeiss LSM 880 Airyscan confocal microscope with a 63× objective lens. Within the Zeiss Zen image acquisition software, the BODIPY FL (488 nm laser) and DAPI (405 nm laser) channel settings were used, as well as T-PMT for DIC.

### Lipid droplet analysis

The CellProfiler software (118) was used to analyze images of BODIPY-stained fungal cells. A pipeline was created to quantify the number of cells, area of each lipid droplet, and number of lipid droplets per fungal cell in a total of 100 cells per sample. The area was defined by the number of pixels within a lipid droplet. The number of lipid droplets per cell was determined by defining lipid droplets that were contained within each parent cell as “child objects.” Three independent biological replicates were used.

### Sterol extraction

Cultures of *C. neoformans* strain KN99 were grown overnight at 30°C in 1× YNB, then subcultured to OD_600_ ~0.03, then grown for 24 h in the presence of DMSO or subinhibitory CPZ concentrations (25 or 50 μg/mL). We then harvested 8 OD units of cells and lyophilized overnight. Samples were resuspended in a mixture of 25% potassium hydroxide/75% EtOH, then incubated at 85°C for 1 h. We added 1 volume of *n*-heptane, vortexed for 3 min, and then analyzed by GC/MS as described (119).

## SUPPLEMENTAL MATERIAL

Fig. S1 msphere.00432-26-s0001.tif Chlorpromazine + AmB increases membrane permeability in *C. neoformans*.

Fig. S2 msphere.00432-26-s0002.tif Transcript levels for other *ERG* genes does not increase with chlorpromazine treatment.

Fig. S3 msphere.00432-26-s0003.tif Chlorpromazine accumulates around lipid droplets.

Fig. S4 msphere.00432-26-s0004.tif Chlorpromazine-sensitive mutants do not increase lipid droplet sizes.

Fig. S5 msphere.00432-26-s0005.tif Wild-type and mutant cells grown in the presence of chlorpromazine or chlorpromazine analogs.

Legends msphere.00432-26-s0006.docx Legends for supplemental figures.

ASM does not own the copyrights to Supplemental Material that may be linked to, or accessed through, an article. The authors have granted ASM a non-exclusive, world-wide license to publish the Supplemental Material files. Please contact the corresponding author directly for reuse.
