# Supplementary material for: Anti-psychotic drugs act synergistically in combination with antifungal drugs to inhibit drug-resistant Cryptococcus neoformans and Candida albicans
Source: mSphere. 2026 Sep 10;11(9):e00432-26. doi: 10.1128/msphere.00432-26 (PMC13621842; doi:10.1128/msphere.00432-26)
Supplement: Supplementary file 1 [file msphere.00432-26-s0006.docx]

**Supplemental Figure 1:** Chlorpromazine + AmB increases membrane permeability in *C. neoformans*.

Chlorpromazine molecular structure showing hydrophilic (circle) and hydrophobic (blue) regions.

**Supplemental Figure 2:** Transcript levels for other *ERG* genes does not increase with chlorpromazine treatment.

**A)** Transcript levels normalized to *ACT1* and then to DMSO-treated cells. Cicles represent biological replicates. Significance calculated using Mann-Whitney test and no differences were identified as significant. **B)** Ergosterol biosynthesis pathway with steps carried out by enzymes encoded by genes tested labeled in blue.

**Supplemental Figure 3:** Chlorpromazine accumulates around lipid droplets.

Cells treated with either vehicle control (DMSO) or chlorpromazine (CPZ) for 1, 2, or 3 hours show accumulation of naturally fluorescent CPZ around the lipid droplets (BODIPY). Scale bar represents 5 μm.

**Supplemental Figure 4:** Chlorpromazine-sensitive mutants do not increase lipid droplet sizes.

Representative cells carrying the labeled mutation were treated with chlorpromazine for 3 hrs, then stained with BODIPY to identify lipid droplets. Scale bar represents 5 μm. Wild-type cells exhibit the characteristic single, large droplet, while chlorpromazine-sensitive mutants mostly do not exhibit enlarged lipid droplets.

**Supplemental Figure 5:** Wild-type and mutant cells grown in the presence of chlorpromazine or chlorpromazine analogs clozapine, perphenazine, or levomepromazine. Consecutive spots represent a serial 5-fold dilution of wild-type or knockout mutant cells. Images from three days of growth at 30°C but were taken one days 2-4. Mutant cells’ resistance to these molecules is visible on days 2-4. Gene name labels are colored to identify ESCRT complex members.
